# Correlation Analysis of Noise, Vibration, and Harshness in a Vehicle Using Driving Data Based on Big Data Analysis Technique

**DOI:** 10.3390/s22062226

**Published:** 2022-03-14

**Authors:** Daehun Song, Seongeun Hong, Jaejoon Seo, Kyounghoon Lee, Youngeun Song

**Affiliations:** 1 Research & Development Division, Hyundai Motor Group, Seocho-gu, Seoul 06797, Korea; song_daehun@hyundai.com (D.S.); seongeun91@hyundai.com (S.H.); hisjj@hyundai.com (J.S.); 9962685@hyundai.com (K.L.); 2 Department of Electrical Engineering, Hoseo University, Asan-si 31499, Korea

**Keywords:** big data, data science, driving data, NVH, correlation analysis, sensitivity analysis

## Abstract

A new development process for the noise, vibration, and harshness (NVH) of a vehicle is presented using data analysis and machine learning with long-term NVH driving data. The process includes exploratory data analysis (EDA), variable importance analysis, correlation analysis, sensitivity analysis, and development target selection. In this paper, to dramatically reduce the development period and cost related to vehicle NVH, we propose a technique that can accurately identify the precise connectivity and relationship between vehicle systems and NVH factors. This new technique uses whole big data and reflects the nonlinearity of dynamic characteristics, which was not considered in existing methods, and no data are discarded. Through the proposed method, it is possible to quickly find areas that need improvement through correlation analysis and variable importance analysis, understand how much room noise increases when the NVH level of the system changes through sensitivity analysis, and reduce vehicle development time by improving efficiency. The method could be used in the development process and the validation of other deep learning and machine learning models. It could be an essential step in applying artificial intelligence, big data, and data analysis in the vehicle and mobility industry as a future vehicle development process.

## 1. Introduction

The importance of driver’s control ability is gradually decreasing in the entire mobility equipment; various commercial vehicles increasingly use new technologies such as advanced driver assistance systems (ADASs), and autonomous driving mobility is also actively being developed through advances in vehicle-to-vehicle (V2V) sensor-data-based positioning technology [1,2]. Although it has not been commercialized, the rapid development of mobility and artificial intelligence technology is expected to cause various changes in many areas, including in daily life in cities. Further, it will affect multiple sectors, such as the social economy and laws in different countries [3,4,5,6].

In particular, the following changes are occurring as the situation changes from one with drivers to a situation in which everyone is a passenger. First of all, devices related to driving will be drastically reduced, indoor space will expand, and various experiences enjoyed while moving will become more important evaluation factors than mobility performance in a wider space. For this reason, technologies related to various user experiences, including infotainment technology, are being actively researched and developed [7].

However, in order for these technologies to be effectively delivered to users, improvement in vehicle comfort and quietness is needed [8]. Therefore, in the design of recent commercial vehicles, noise, vibration, and harshness (NVH) are evaluation factors for vehicle design and development. NVH is the technical term used in measuring and modifying the noise and vibration characteristics of vehicles. Ears hear noise, and passengers’ various body parts feel the vibration. Harshness includes both but is related to a feeling of roughness. NVH indicates the numerical degree of factors constituting ride comfort, such as interior noise and body vibration of the vehicle. It is higher than ever and is expected to increase further in the future.

In order to reduce the interior noise and vibration of a vehicle, it is most important to identify the excitation source or noise source that has a large contribution to the noise and vibration in the problem frequency band. In particular, as the driving part of automobiles has recently changed from combustion engines to electric motors, parts such as the exhaust system have disappeared, a large number of new parts such as batteries have been added, and structures have been greatly changed. The demand for accurate NVH analysis technology is growing more than ever [9,10,11].

Noise generation and transmission of vehicles related to NVH can be divided into small pressure waves moving into the air and noise caused by vibration through the vehicle structure. In the former case, for example, the sound is physically emitted from an exhaust pipe and transmitted. In the latter case, acoustic energy generated from a vibration source such as an engine moves through the rigid vehicle body and then flows into the interior as noise and vibration or is generated by road stimuli transmitted through tires and suspension systems [12,13]. As such, noise and vibration form a complex relationship, and nonlinear elements act strongly. Thus, the main goal of an NVH experiment is to analyze various types of vibration and noise characteristics generated by the vehicle and the route transmitted to the driver and passenger as experimental data [14].

In most NVH analysis methods, a physical/statistical approach is generally used to check the frequency response function (FRF) or noise transfer function (NTF) from a suspicious area to a target (noise or vibration). Transfer pass analysis (TPA) analysis is also used. The structural and vibrational sound transfer function of FRF still plays an essential part in NVH data analysis in vehicle development programs. Knechten et al. [15] proposed a method of attaching a sensor to obtain data in three directions to obtain all relevant FRFs inside and outside a vehicle body structure. They proved their method’s efficiency through an experimental evaluation. This method can successfully obtain the result of NVH analysis through FRF under specific experimental environment conditions, but it is limited in terms of comprehensive NVH analyses through various sensor data in various driving situations.

A study using a driving simulator was conducted by Sturm et al. [16] in order to efficiently obtain data for analyzing NVH, using the vehicle noise transfer function NTF in various driving environments. The use of a driving simulator has the advantage of being able to study complex situations that can occur safely in a vehicle with high repetition. Through this NVH simulation, it is possible to sufficiently verify the suitability of parts and structures before mounting a vehicle, but there is a point where there is inevitably a difference from the actual driving NVH result of the vehicle. In addition, whenever the vehicle structure or other part changes, the simulator must be reconfigured, and data must be acquired again [17].

It is difficult to accurately reflect some factors in the NVH analysis with this technique. This is due to the complexity of vehicle parts, the dynamic characteristics at a specific frequency change according to the excitation range, and the nonlinearity that occurs when the frequency of a specific mode changes. Therefore, a statistical approach has recently been attempted for the statistical quantification of the distribution of vehicle NVH data and analysis of the cause of the distribution.

Researchers such as Dowsett [18] have compared and analyzed NVH data from a statistical point of view of the response characteristics at various points of various vehicles. As a result, a method was proposed for making NVH proximity prediction data using vehicle-to-vehicle variability data. The data used in the experiment result from measuring the impact response by an impact hammer, but there is a need to confirm these results using various environmental and sensor data. Hills et al. analyzed the causes of NVH data obtained from hundreds of different types of vehicles through statistical quantification in subdivided areas [19].

However, it is difficult to find a case of actively using NVH data distributions for correlation and sensitivity analysis, and there are no cases that use vehicle NVH big data, as undertaken in this study. Therefore, it is a challenging task for NVH researchers to find the exact areas that need improvement the most, and they consider simple driving data and FRFs due to the complexity of vehicle NVH and time-consuming tasks. It is even more difficult to investigate how much internal noise increases when a system vibration or noise occurs, and how much a system vibration or noise level needs to be reduced to meet an acceptable interior noise level.

This study proposes a novel big data-based correlation analysis technique to contribute the following features to the field of NVH analysis used in vehicle development:

- Intuitive and simply numerical analysis of the correlation and sensitivity of each vehicle part;
- Possible to plan a more accurate selection plan of development goals considering NVH improvement.

We propose a technique that intuitively and simply numerically analyzes the correlation and sensitivity of each measurement part to a target using only driving data under various conditions. These data can be used instead of performing additional experiments or creating a specific model. A more accurate selection plan for development goals is also presented. Thus, by performing the proposed process, researchers can find areas that need improvement through correlation analysis and variable importance analysis, and determine how much room noise increases when the NVH level of the system changes through sensitivity analysis. Additionally, by performing a system target analysis, a system NVH level goal can be selected. Accurate analysis of sensitivity and selection of development goals based on data can shorten vehicle development time by improving the accuracy of tuning and test efficiency in the development stage. The analysis can also be used as a criterion in the design stage. It is an essential step in the early stages of development. The technique proposed in this study applies data analysis techniques to vehicle NVH sensitivity analysis and could be useful in the future.

The NVH data obtained for the performance evaluation of the proposed method are explained in Section 2. In Section 3, data analysis results are described in detail using EDA, an variable importance analysis is provided using machine learning, correlation analysis, and sensitivity analysis. In Section 4, contents that could contribute to the selection of development goals using technical statistics are explained, and finally, conclusions and discussion of future research directions are presented in Section 5.

## 2. NVH Big Data for Random Driving Situation

**Figure 1:**
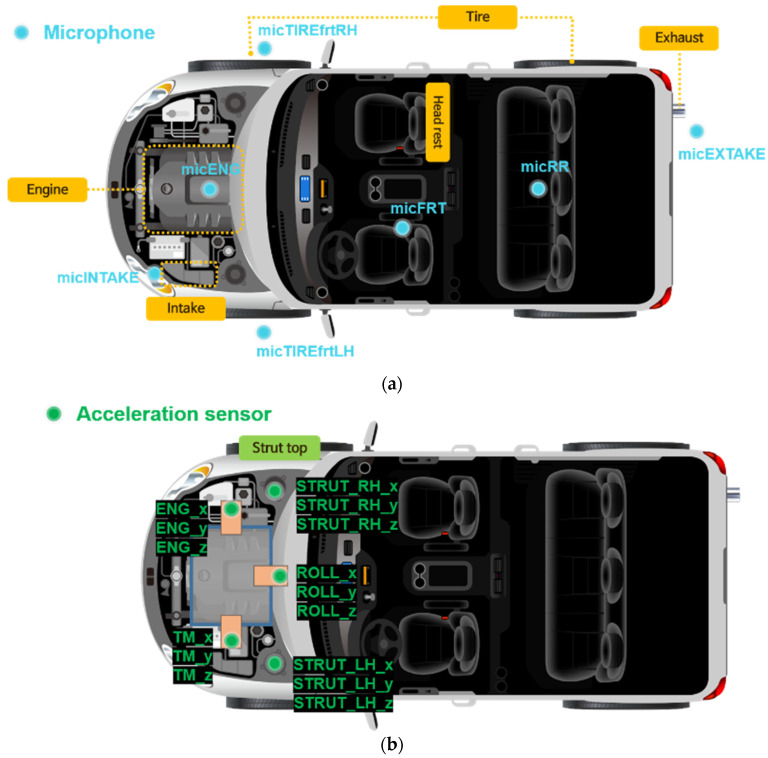
Sensor positions on a vehicle with variables for data acquisition: (**a**) microphone; (**b**) acceleration sensor.

**Figure 2:**
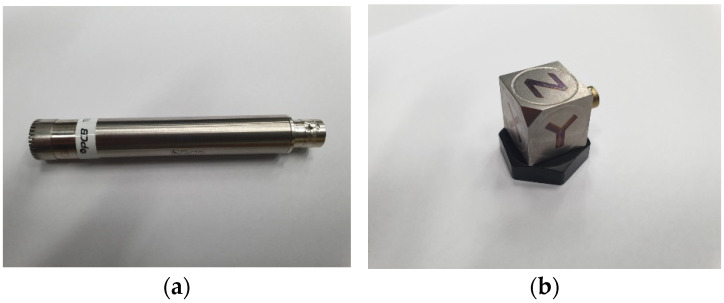
Sensors used in experiments: (**a**) microphone; (**b**) acceleration sensor.

In order to acquire noise and vibration data in a random driving situation, seven microphones and five three-axis accelerometers were installed in the main parts of a vehicle, as shown in Figure 1. The vehicle information used in the experiment is shown in Table 1. The system was constructed so that a total of 22 signals can be acquired in real time when driving by combining 7 sets of sound data obtained from the microphones and 15 sets of vibration data. Each and every 22 sensor signal data point was acquired every 0.5 s at 1 Hz intervals, from 1 to 500 Hz. Therefore, 1,320,000 signals were obtained every minute. To measure the noise source, data from multiple microphones installed in the engine, intake, exhaust, and tires were measured, and a three-axis accelerometer was used to measure the vibration generated from the excitation source. Data from the engine, transmission, strut, and center of the vehicle chassis were measured. In addition, by attaching microphones indoors, changes in indoor noise heard by the driver and passengers in the rear seat were measured and recorded in real time. The specifications of the microphone and acceleration sensor used in the experiment are shown in Table 2, and Figure 2 shows the actual images.

**Figure 3:**
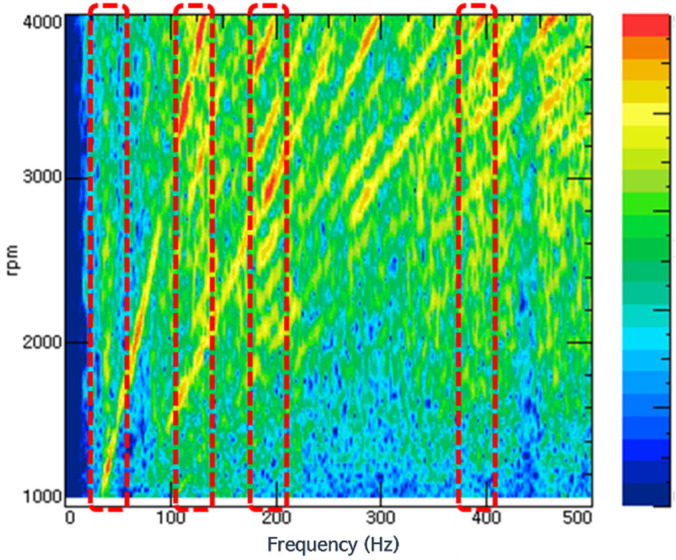
Indoor noise in frequency domain during rapid acceleration; dotted red line indicates main frequency band of interest.

In order to accurately analyze sensitivity, it is necessary to understand the characteristics and phases of the transmission functions of various transmission paths and excitation sources. The characteristics of vehicles with numerous resonance modes, sound field modes, and complex nonlinear dynamic characteristics must be accurately identified. Figure 3 shows the change in indoor noise in the frequency domain area during rapid acceleration of a sedan vehicle by representing initial driving data. In the NVH development phase, determining acceleration NVH performance based on data related to rapid acceleration conditions is essential. In particular, the main frequency band of interest is the area indicated by the dotted red line in Figure 3, which is a frequency band that includes a section with high sound pressure, compared with other areas. Drivers and passengers decide the main frequency band of interest. First, when several noises were judged to be declined by test drivers, interior noise was measured, and NVH engineers differentiated the noises into several problematic frequencies. Even though high sound pressure was measured in a specific frequency band, it could be neglected if it was covered by other noise or did not annoy passengers. The sensitivity of each excitation source in that frequency band becomes an important issue in the stage of vehicle development.

**Figure 4:**
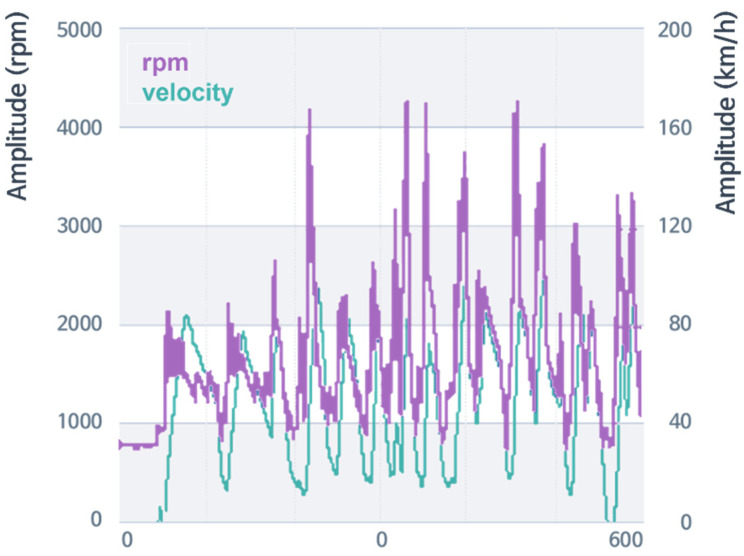
RPM and vehicle velocity data example (the raw data of each sensor is in the time domain) under arbitrary driving conditions.

RPM ranged from 800 to 4200, and velocity ranged from 0 to 100 kpm. Vehicle velocity was suggested to be lower than 80 kph overall to neglect the wind noise effect. The measurement was carried out on the proving ground in the Hyundai-Kia R&D center. The primary excitation sources were powertrain and road. The sensor points were on the main points of the vehicle body, excited by the primary excitation sources. In order to obtain data for sensitivity analysis by frequency, data under random driving conditions were used for analysis, as shown in Figure 4. Although most of the analysis was conducted in the frequency domain after performing FFT, the raw data of each sensor were in the time domain, and all the CAN data from vehicle buses were also in the time domain. They are expressed in Figure 4. For the purpose of avoiding analysis by only excitation and response in a specific area, random driving was performed to acquire data in various situations that a vehicle may experience during driving. In addition, in order to avoid excessive wind noise, a vehicle was driven at 90 km/h or less, and data measured for more than 15 min were used for this analysis.

**Figure 5:**
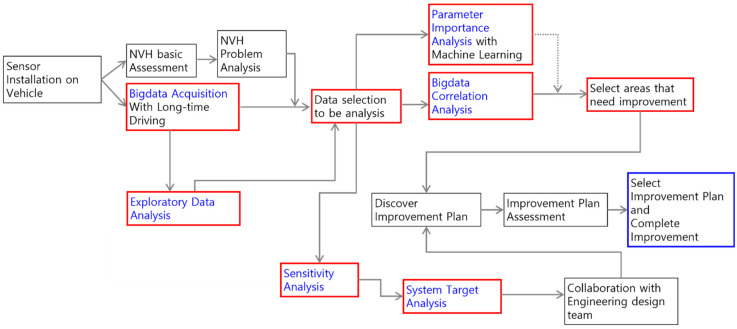
The NVH analysis flowchart based on driving data.

Figure 5 shows the proposed NVH analysis method flowchart. In this study, driving data of excitation sources (powertrain vibration/noise, suspension vibration, intake/exhaust noise, tire noise) and targets (indoor noise/vibration) were obtained in various driving conditions (various road surfaces and vehicle speeds). After that, the sensitivity ranking was qualitatively and quantitatively calculated by obtaining the distribution and correlation coefficient between the excitation source and the target in a specific frequency band. In the data analysis, the preprocessed data were used to match the entire frequency domain data that had undergone a fast Fourier transform (FFT) for each excitation source and target at each frequency. The preprocessing technique was the same as the one used in a frequency-based spectrum prediction model when predicting vehicle interior noise and vibration based on driving data using deep learning [20].

## 3. Data Analysis Method

### 3.1. Exploratory Data Analysis

EDA originated from the first use by scholar John Tukey in his book published in 1977. It refers to an analysis technique that grasps the overall structure and relationship of data through basic statistical techniques and various visualizations [21]. First, the process of grasping the data structure as a whole through several visualizations should always precede data analysis. EDA is important to check that the data have no problem and show Gaussian distribution [22]. If the data offer different ranges or distributions from those of the normal data, the data would be neglected. Additionally, if the data are far from Gaussian distribution, data filtering is carried out, separating the data according to different conditions. In this study, R was used for data analysis. R is a set of computer languages and packages that support statistics, machine learning, and graphics.

Statistical methods and machine learning were used to find the solution for some NVH problems. To obtain enough data to analyze, multisensor measures during continuous long-time driving, and the time-domain data were transferred to the frequency domain. The areas that need improvement were selected through the correlation analysis and parameter importance analysis. Sensitivity analysis and system target analysis resulted in suggestions on the improvement needed. These two essential pieces of information help engineers to develop improvement plans and complete improvement.

**Figure 6:**
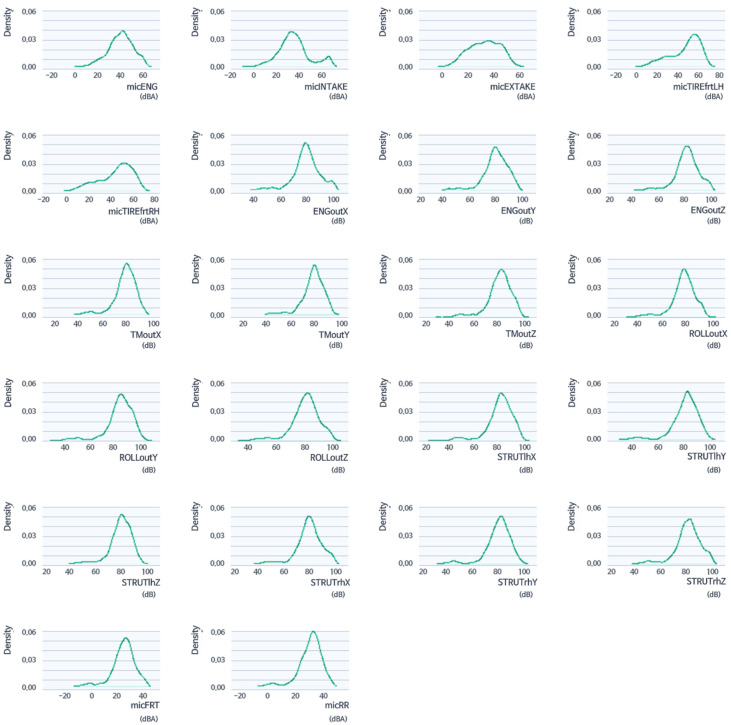
Density distribution of variables (42 Hz) has a normal distribution, under random driving conditions.

In this study, an in-depth analysis of booming noise was carried out in the low-frequency band area, which has the most crucial influence on drivers and passengers among indoor noise. Although different for each vehicle characteristic, booming noise, including lock-up booming noise, mainly occurs around 25–50 Hz [23,24]. In this system, the booming noise band was designated as the frequency band of interest (42 Hz), and the data were analyzed. As shown in Figure 3, this process was performed because the corresponding band was the first vertical acoustic mode that appeared in the frequency domain and therefore the main control target. The density distribution of the values of each variable was examined to examine whether the data received under the random driving conditions had a normal distribution. Figure 6 shows the density distribution, and it can be seen that it generally showed a normal distribution. In addition, in order to examine the overall relationship between data in the 42 Hz band, one variable was paired with the other variables, and scatter plots in a grid format were arranged in one graph.

**Figure 7:**
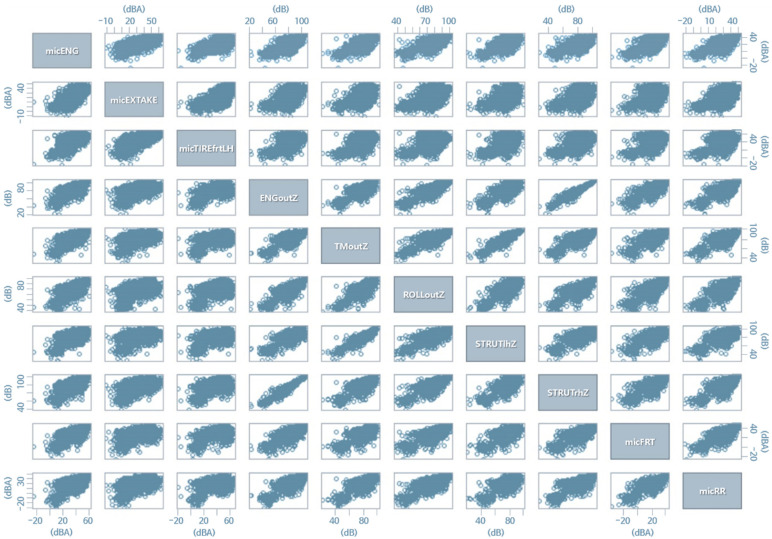
Scatter plot between 10 of the totals of 22 variables used in the analysis to examine the overall relationship (42 Hz).

Figure 7 shows a scatterplot with only 10 of the total of 22 variables used in the analysis in the 42 Hz band. It provides insight into the overall structure and correlation of the data to be analyzed through this visualization. It is possible to intuitively detect a strong linear correlation between the vertical vibration of the engine mount body side and the vertical vibration of the right side strut. EDA was evaluated to determine how the entire data were distributed, and how the systems were correlated. If we had a considerable volume of data, and some points showed very strong correlations, we had to consider reducing those data to increase computational efficiency. The correlation analysis and variable importance analysis reveal that the engine mount and strut were important points regarding interior noise in the 42 Hz band. Additionally, the EDA results indicate that both were correlated; therefore, if one area is improved, it might show enough improvement effects overall since the two points were relatively close geometrically.

### 3.2. Variable Importance Analysis Using Machine Learning

Prior to the full-scale analysis, we applied the given data to a random forest model, one of the formal data mining techniques, and analyzed the importance of input variables for target variables. Random forest is the ensemble model of the decision tree model [25,26]. It could diminish problems, compared with other machine learning models. The prediction method should use validation data separately from training data to ensure that those effects are negligible. The software used was implemented through the R programming language, and the random forest library was used. After modeling a random forest model with 500 trees, variable importance was selected. The dataset was 22 × 2093, and there were 21 input channels and 1 target channel. Random forest is an ensemble model of a decision tree model used to classify groups or perform predictions by representing decision rules in a tree structure. It is a widely used technique in machine learning due to its excellent accuracy [27]. There is an existing case of predicting and correlating idle booming and vehicle vibration using deep learning and machine learning [28].

**Figure 8:**
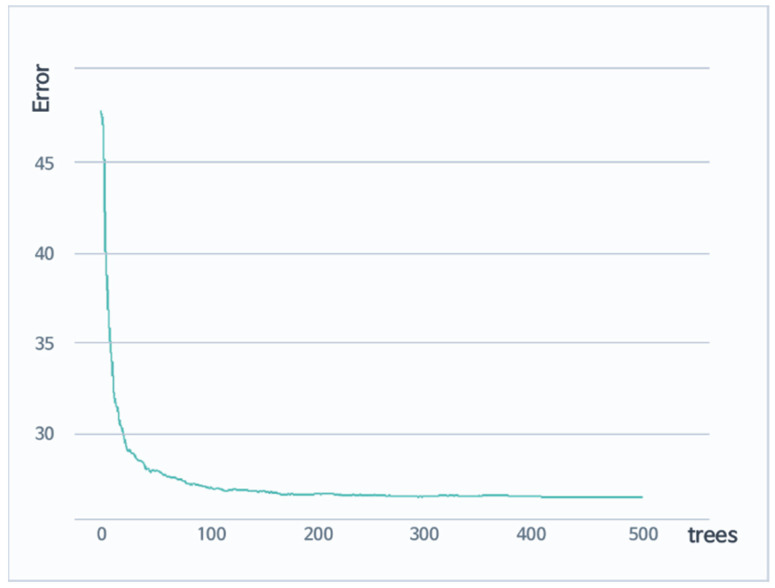
Sum of the residual squares of the trained model. The model converged as the number of ensembled trees exceeded 400.

In this study, when all seat noise was targeted, and the remaining variables were input, a model was constructed with a random forest. Figure 8 shows the sum of the residual squares of the trained model. It can be confirmed that the model converged, as the number of ensembled trees exceeded 400.

**Figure 9:**
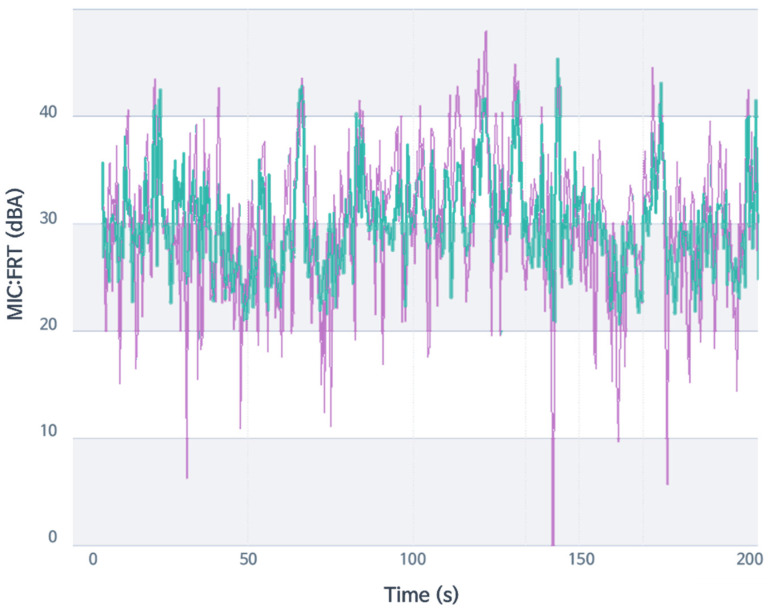
Comparison of test data results: violet, the actual data; green, the predicted result.

**Figure 10:**
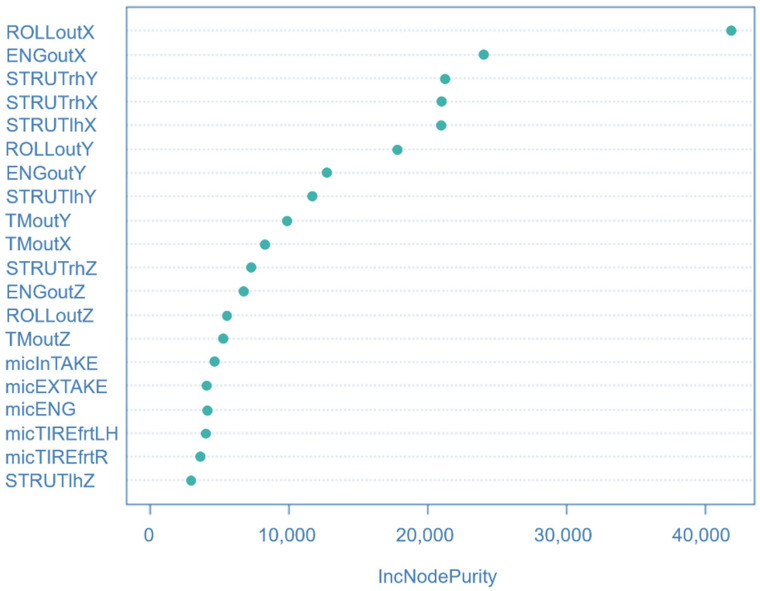
Variable importance. In the 42 Hz band, it is inferred that the forward and backward vibration of the roll rod body is the most important variable for seat noise.

The verified results of the test data can be confirmed in Figure 9, in which the blue color shows the actual data, and the green color shows the predicted result. Generally, the excellent predictive performance was confirmed. Figure 10 shows a graph of the importance of the variables obtained as a result of model analysis, and it was found that the front–rear vibration of the roll rod body was the most important variable for all seat noise in the 42 Hz band. Variable importance analysis was used to support the correlation analysis results. By comparing variable importance analysis and correlation analysis, researchers can decide on the system or area to be improved. This method using machine learning needs to be further improved by future research.

### 3.3. Correlation Analysis

**Figure 11:**
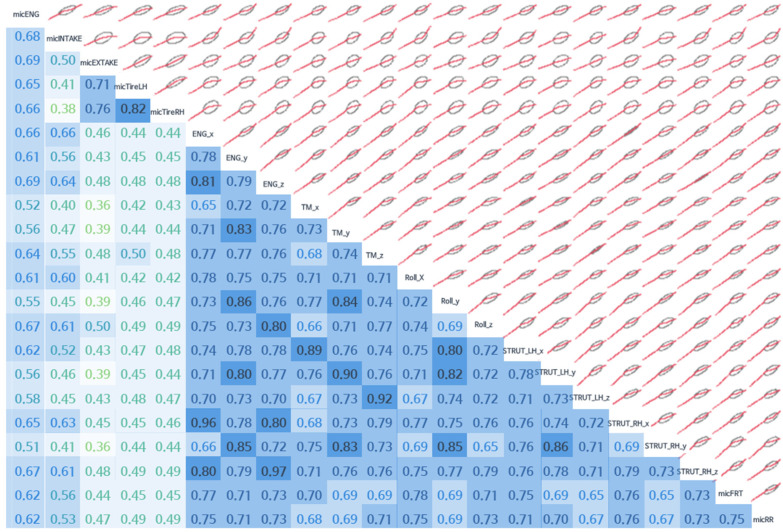
Correlogram (42 Hz) between variables.

As shown in Figure 11, more information can be discerned at the same time by showing the distribution, linearity, and correlation between variables on one side and a correlogram on the other. A correlogram is a graph of the correlation coefficient. The larger the correlation coefficient, the darker the value is. The ellipse shows the approximate distribution of each data point, which helps with an intuitive understanding of the data [29].

**Figure 12:**
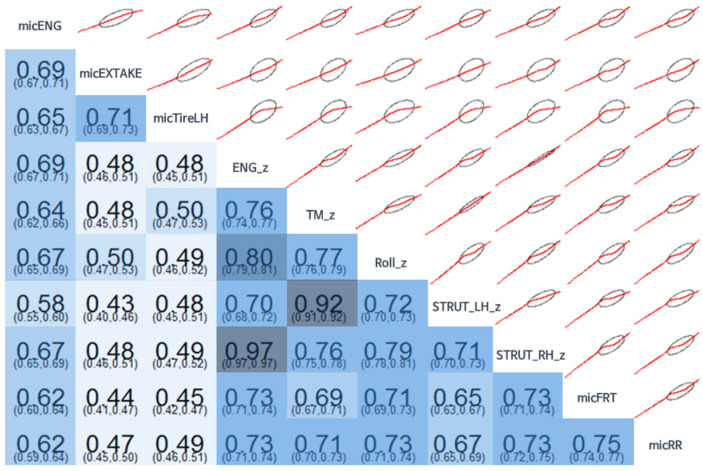
Correlogram (42 Hz) between 10 selected variables related to the z-direction. The z-direction data were regarded as the most important in vehicle NVH data.

For Figure 12, only 10 variables were selected for better visibility (these 10 representative variables were selected since the data on the z-direction were regarded as the most important, compared with the x- and y-direction in vehicle NVH data), which are displayed under a correlation coefficient up to the 95% confidence level range of the correlation coefficient, in the same form as in Figure 11. The Pearson correlation coefficient was used for the correlation coefficient. The coefficient equation used in the calculation is shown in Equation (1).

(1) $$\rho \left(X,Y\right)=\frac{cov\left(X,Y\right)\;}{\sigma_{X}\sigma_{Y}}$$

In Table 3, the correlation coefficients between the different variables and all seat noise in the 42 Hz band are listed in the order of highest correlation coefficient. It can be seen that they were somewhat similar to the ranking of variable importance. In addition, Table 4 lists the correlation coefficients between the different variables and all seat noise in the 397 Hz band in the order of highest correlation coefficient. In consideration of the difference in response characteristics between noise and vibration, the noise source and the vibration source were separated and ranked. It can be seen that there was a difference in the ranking of the correlation coefficient for each frequency, and there was a slight difference in the average size of the correlation coefficient.

Judging from the shape of the scatter plot and the correlation coefficient, in the sedan vehicle used in the test, the front–rear direction (Roll_x) and the front–rear direction of the strut (Strut_LH_x, Strut_RH_x) had relatively large correlations in the 42 Hz band of front seat noise. It can be determined that the exhaust system noise and tire noise had relatively weak correlations. This analysis allows the user to change the desired target in a specific frequency (e.g., 42 Hz) and a specific frequency range (e.g., 40 to 45 Hz) in various ways, such as front–rear seat noise and indoor floor vibration. The analysis also makes infinite analysis possible. In the 397 Hz band, it can be seen that the correlation between the strut vibration was high, while the correlation between the engine room noise was relatively low.

**Figure 13:**
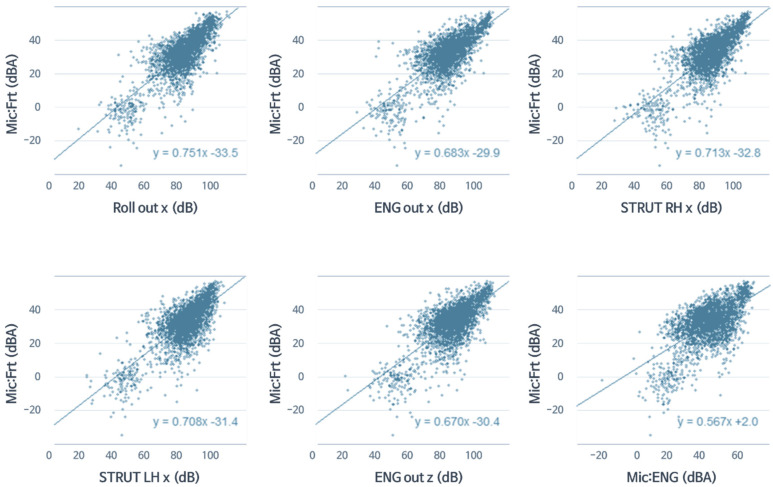
Linear regression of all seat noise with highly correlated variables in the 42 Hz band.

To avoid the difference in results from various machine learning methods at this phase, this research focused on the correlation analysis result obtained from the sensor data themselves. Following sensitivity analysis and selection of development, goals were developed based on the correlation analysis results. Therefore, Figure 13 reflect up to the fifth rank in vibration sources and first rank in noise sources from the correlation results, as shown in Table 3.

### 3.4. Sensitivity Analysis

After determining the linearity and correlation of the data, it was possible to statistically find the degree to which the response changes when each excitation source changes through linear regression. Figure 13 shows the result of linear regression of all seat noise with variables with high correlations in the 42 Hz band. Each excitation source (ROLL_x, ENG_x, STRUT_RH_x, STRUT_LH_x, ENG_z, engine room noise) is on the *x*-axis, the *y*-axis is all seat noise, and each linear regression line and sensitivity are expressed as slopes.

When ROLL_x vibration increased by 1 dB, the noise of all seats could be statistically expressed as an increase of 0.751 dBA. Therefore, the sensitivities of the six variables (ROLL_x, ENG_x, STRUT_RH_x, STRUT_LH_x, ENG_z, engine room noise) with strong correlation at 42 Hz were 0.751, 0.683, 0.713, 0.708, 0.670, and 0.567, respectively. Thus, it can be seen that there was a slight difference between the ranking of the correlation and the ranking of the sensitivity.

**Figure 14:**
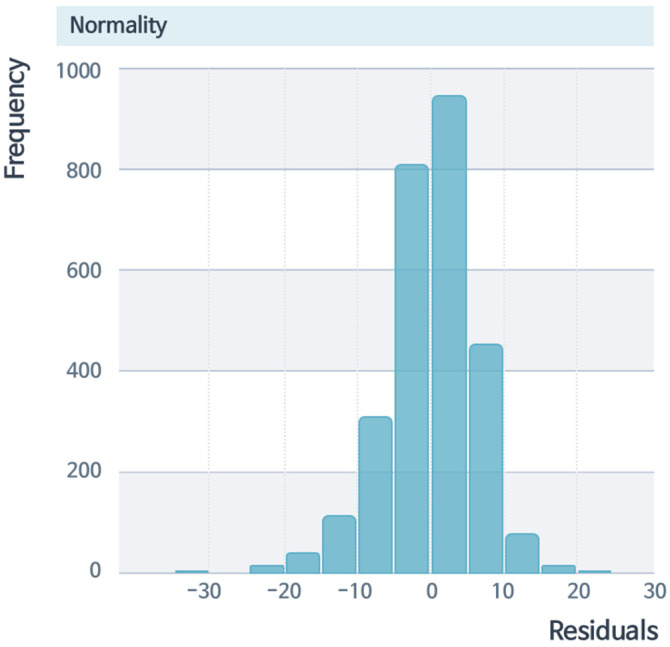
Normality of linear regression residuals. The normality condition was satisfied with a normal distribution shape.

**Figure 15:**
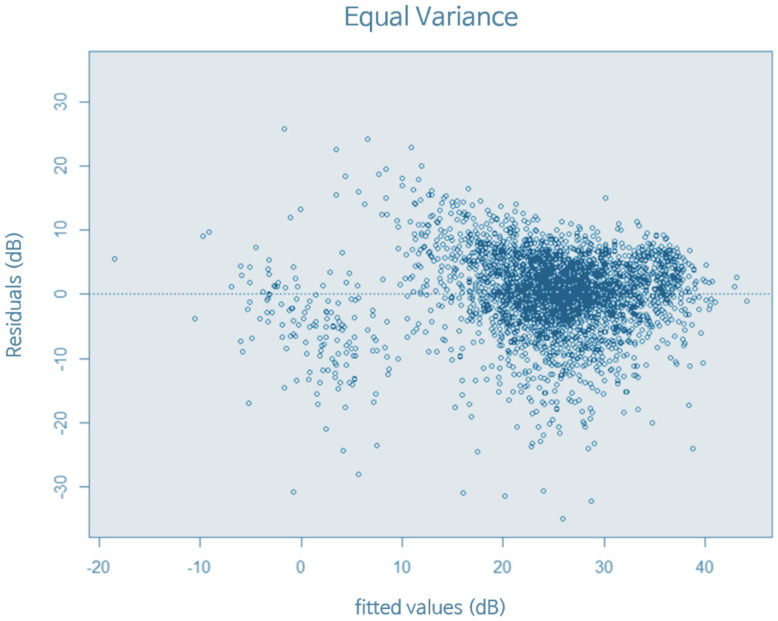
Equal variance of linear regression residuals. The data show generally well distributed up and down.

Regarding the suitability of the linear regression model, Figure 14 shows the normality of the linear regression residuals. The frequency on the vertical axis in Figure 14 represents the number of frequencies for how often the data occurred [30]. The shape was generally in a normal distribution, and the normality condition was satisfied. Figure 15 shows a graph expressing the equal variance of the residuals. It can be seen that they were generally well distributed up and down, without bias.

## 4. Selection of Development Goals Using Technical Statistics

Descriptive statistics were used to express the overall composition of data in simple numbers. The median, average, highest, lowest, first quartile, and third quartile are represented by descriptive statistics. In this study, the median number and the first quartile were harmful when selecting the development goals of each variable within a specific target (in this study, all seat noise). Therefore, the third quartile was used. The median is the value in the middle rank in the total data, the first quartile is the value in the top 75%, and the third quartile is the value in the top 25%. The difference between the third and first quartiles is also referred to as the interquartile range (IQR).

**Figure 16:**
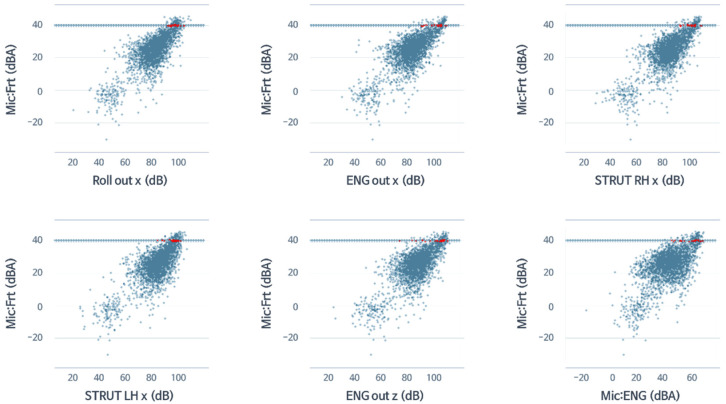
Sampling of data of highest correlation coefficient six areas for each variable at 40 dBA (±0.5) of all seat noise in the 42 Hz band: red indicates variables with a high correlation.

**Figure 17:**
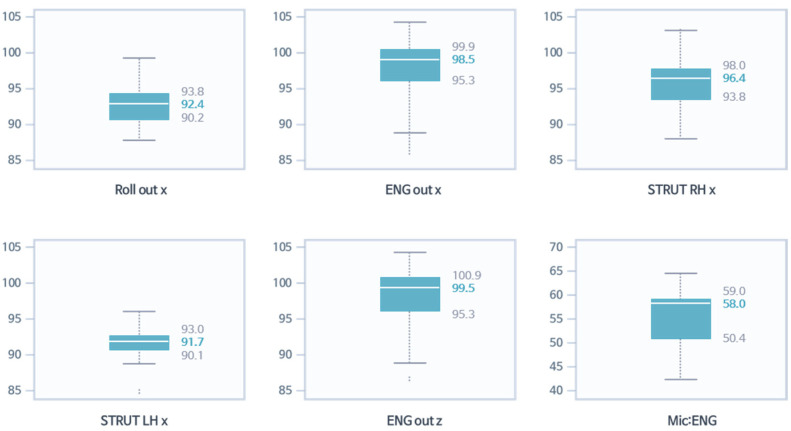
Selection of development targets for each variable for 40 dBA of all seat noise in the 42 Hz band. The quartiles can be used to select development targets for each variable.

In Figure 16, assuming that the development target is 40 dBA for all seat noise in the 42 Hz band, only data within the range of ±0.5 at 40 dBA were selected. They are expressed in red for variables with high correlation. The selected six areas were selected as the area with the highest correlation coefficient among all areas with reference to Figure 10 and Table 3. Figure 17 shows the data adopted in Figure 16 as a box plot to show the quartiles at a glance.

The quartiles shown in Figure 17 can be used to select development targets for each variable. Moderate targets were chosen in the median, aggressive targets were chosen in the lower third quartile, and defensive targets were chosen in the first quartile and were slightly higher than the median. Therefore, the goal for ROLL_x was 92.4, but a conservative goal of 90.2 could be selected, and a defensive goal could be 93.8. This selection method could play a significant role in cooperation with design and analysis in a test by statistically representing the development goals of the variables for the desired target in the desired frequency band at once after driving evaluation.

During the vehicle development phase, it is important for the test department to provide detailed test information when improving the NVH in collaboration with other departments such as project management or ride and handling (R&H) departments. Knowing the extent to which internal noise is improved when a particular system or area of a vehicle is improved is very important for changing and improving vehicle design and system performance. Therefore, if certain parts of the system are not correlated with NVH performance, then correlation analysis is paramount because there is no need to change the system. Additionally, system target analysis and sensitivity analysis are essential for sophisticated engineering, which was challenging and inaccurate with conventional methods. Additionally, a target value of 40 dBA (±0.5) was selected for this study only. When the target value is changed, the results vary. The target value and the range (±0.5) affect the results. NVH engineers’ development target would decide the target value. The target and scope are determined according to the vehicle class and development concept, and the target value is set differently according to such cases.

## 5. Conclusions

In this study, we used NVH driving data—all real-time spectral data were longer than 15 min—from a long period in a random driving situation, for the 42 Hz bandwidth, which is the booming noise range of indoor noise focusing on acceleration NVH performance. Through the process of selecting a development goal, useful results were confirmed by applying data analysis and machine learning techniques in the vehicle development stage for NVH. The data analysis technique using all of these data reflected all the parts that could not be explained by the existing analysis of specific order components and the analysis of only FRF, which did not reflect the nonlinearity of dynamic characteristics. Our method analyzed the data without wasting the measured data. Unlike existing methods such as FRF, NTF, and TPA, multisensor data were comprehensively analyzed in various actual driving situations, and development period and cost could be dramatically reduced. It can be claimed that this technique reflects the next-generation trend of big data techniques. Using the proposed method, through the actual driving noise and vibration data, it is possible to determine the connectivity between interior noise and the various system NVH factors through correlation analysis and variable importance analysis.

From the example shown in this research, engineers can infer that the 42 Hz interior noise of the seat of the driver is highly related to the front–rear vibration from the roll rod on, especially acceleration condition. The sensitivity of the interior noise of the seat of the driver to the vibration from the roll rod was 0.751. To reduce the interior noise level to 40 dBA in the 42 Hz band, the front–rear vibration from the roll rod was suggested to be less than 92.4 dB.

As an analysis method that prevents overfitting and distortion of the generated model due to the creation of a new model, it intuitively and numerically represents the data. In addition, it could be useful both for sensitivity analysis and comparative evaluation of contributions in various environments when an improvement sample is applied. In the future, the same experiment will be repeated in all frequency ranges to evaluate and analyze the proposed technique. We plan to expand the technique by expanding it to various vehicle types, analyzing the differences in results and security points, and performing NVH analysis using big data. In addition, this technique is expected to be useful as a direct application in the development process, as well as a verification technique for verifying models using deep learning and machine learning in the future. It is expected to become an important model for next-generation vehicle development. In addition, the proposed method is not limited to specific conditions such as commercial vehicle development, and it is expected to make an innovative contribution to the industry as a technology that can be applied immediately to all mobility fields. In particular, we plan to apply the technology to a delivery robot that is sensitive to vibration in the future. Factors affecting the vibration of loads will be analyzed and applied to develop a robot capable of safe and effective delivery. In addition, we plan to continue research on efficiently reducing noise pollution from robots by analyzing the causes of noise generated by robots with the proposed technique.

## Figures and Tables

**Table 1:** Vehicle specifications used in the experiment.

Body	Mid-Size Sedan
Weight	1464 kg
Engine	1.6 L inline 4 gasoline engine with turbocharger
Front suspension	MacPherson strut
Rear suspension	Multi-link

**Table 2:** Sensor specifications.

Sensor	Type	Frequency Range	Detailed Description
Microphone	378B02	3.75–20 kHz	1/2″ prepolarized free-field condenser microphone
Acceleration sensor	356A15	2–5 kHz	triaxial, high sensitivity, ceramic shear accelerometer

**Table 3:** The 42 Hz band excitation/noise source vs. driver’s seat noise correlation coefficient and its ranking.

Rank	1	2	3	4	5	6	7	8	9	10
Variable	Roll_x	ENG_x	Strut_RH_x	Strut_LH_x	ENG_z	Strut_RH_z	ENG_y	Roll_z	TM_x	TM_y
Correlation coefficient	0.783	0.765	0.763	0.750	0.727	0.725	0.715	0.710	0.695	0.691
Rank	11	12	13	14	15	Noise source 1	Noise source 2	Noise source 3	Noise source 4	Noise source 5
Variable	TM_z	Strut_LH_y	Roll_y	Strut_RH_y	Strut_LH_z	micENG	micINTAKE	micTireRH	micTireLH	micEXTAKE
Correlation coefficient	0.691	0.687	0.686	0.650	0.650	0.623	0.562	0.451	0.446	0.440

**Table 4:** The 397 Hz band excitation/noise source vs. driver’s seat noise correlation coefficient and its ranking.

Rank	1	2	3	4	5	6	7	8	9	10
Variable	Strut_LH_y	Roll_y	Strut_LH_x	Strut_RH_x	Strut_RH_z	Strut_LH_z	TM_x	Strut_RH_y	Roll_z	ENG_y
Correlation coefficient	0.811	0.783	0.770	0.755	0.740	0.733	0.704	0.701	0.700	0.695
Rank	11	12	13	14	15	Noise source 1	Noise source 2	Noise source 3	Noise source 4	Noise source 5
Variable	Roll_x	ENG_x	ENG_z	TM_y	TM_z	micTireLH	micTir-eRH	micEXTAKE	micIN-TAKE	micENG
Correlation coefficient	0.687	0.669	0.663	0.632	0.626	0.674	0.649	0.604	0.573	0.538
